# The role of climate, vegetation, and soil factors on carbon fluxes in Chinese drylands

**DOI:** 10.3389/fpls.2023.1060066

**Published:** 2023-02-09

**Authors:** Zhaogang Liu, Zhi Chen, Guirui Yu, Weikang Zhang, Tianyou Zhang, Lang Han

**Affiliations:** ^1^ Key Laboratory of Ecosystem Network Observation and Modeling, Institute of Geographic Sciences and Natural Resources Research, Chinese Academy of Sciences, Beijing, China; ^2^ College of Resources and Environment, University of Chinese Academy of Sciences, Beijing, China; ^3^ Yanshan Earth Critical Zone and Surface Fluxes Research Station, University of Chinese Academy of Sciences, Beijing, China; ^4^ College of Grassland Agriculture, Northwest A&F University, Yangling, China; ^5^ Institute of Surface-Earth System Science, School of Earth System Science, Tianjin University, Tianjin, China

**Keywords:** carbon flux, dryland, grassland, climate change, vegetation, soil

## Abstract

Drylands dominate the trend and variability of the land carbon (C) sink. A better understanding of the implications of climate-induced changes in the drylands for C sink-source dynamics is urgently needed. The effect of climate on ecosystem C fluxes (gross primary productivity (GPP), ecosystem respiration (ER), and net ecosystem productivity (NEP)) in drylands has been extensively explored, but the roles of other concurrently changing factors, such as vegetation conditions and nutrient availability, remain unclear. We used eddy-covariance C-flux measurements from 45 ecosystems with concurrent information on climate (mean annual temperature (MAT) and mean annual precipitation (MAP)), soil (soil moisture (SM) and soil total nitrogen content (soil N)), and vegetation (leaf area index (LAI) and leaf nitrogen content (LNC)) factors to assess their roles in C fluxes. The results showed that the drylands in China were weak C sinks. GPP and ER were positively correlated with MAP, while they were negatively correlated with MAT. NEP first decreased and then increased with increasing MAT and MAP, and 6.6 °C and 207 mm were the boundaries for the NEP response to MAT and MAP, respectively. SM, soil N, LAI, and MAP were the main factors affecting GPP and ER. However, SM and LNC had the most important influence on NEP. Compared with climate and vegetation factors, soil factors (SM and soil N) had a greater impact on C fluxes in the drylands. Climate factors mainly affected C fluxes by regulating vegetation and soil factors. To accurately estimate the global C balance and predict the response of ecosystems to environmental change, it is necessary to fully consider the discrepant effects of climate, vegetation, and soil factors on C fluxes, as well as the cascade relationships between different factors.

## 1 Introduction

Drylands are areas where precipitation is scarce and usually unpredictable and generally refer to areas where the aridity index (AI) is lower than 0.65. Drylands cover 41% of the global land surface (Feng and Fu, 2013) and sustain 38% of the world’s population (Koutroulis, 2019). China is one of the largest drylands in the world (Prăvălie, 2016). Among the all terrestrial ecosystem types, drylands have the most fragile ecological environments and are most sensitive to environmental change (Yao et al., 2020). The change in land atmospheric carbon (C) exchange can cause distinct fluctuations in atmospheric CO_2_ concentrations and thereby affect global climate change (Piao et al., 2019). Grasslands, shrublands, and savannas are the primary ecosystems of drylands and are considered to be the main sources of interannual variations in the global land C sink (Poulter et al., 2014; Ahlstrom et al., 2015). The large interannual variation is mainly caused by dryland response to the interannual fluctuations in precipitation (Knapp et al., 2015). At the same time, the warming and drying trends predicted in the future may also cause dramatic changes in the structure and function of dryland ecosystems (Berdugo et al., 2020). A better understanding of the implications of climate-induced changes in dryland ecosystems under future climate scenarios for C sink-source dynamics is urgently needed.

Many studies have shown that drylands perform a distinct C absorption function (Dong et al., 2011), but other studies have noted that the C absorption capacity of drylands is limited, and their weak C source or sink characteristics are prone to directional reversal under the influence of climate factors (Flanagan et al., 2002; Biederman et al., 2017). Gross primary productivity (GPP), ecosystem respiration (ER), and net primary productivity (NEP) were reported to be positively correlated with mean annual temperature (MAT) and mean annual precipitation (MAP) across different ecosystems (Law et al., 2002; Yi et al., 2010). Within a wider climate zone, the relationship between these climate factors and C fluxes (GPP, ER, and NEP) was relatively strong in Asia (Hirata et al., 2008; Kato & Tang, 2008; Yu et al., 2013). In China, the spatial patterns of GPP, ER, and NEP are determined by MAT and MAP, and these response patterns are consistent in different ecosystems. Precipitation is reported to be the most important limiting factor in drylands, and dominates the spatial-temporal pattern of change in productivity (Bernacchi and VanLoocke, 2015). As the main water source, precipitation not only affects photosynthesis, but also affects autotrophic and heterotrophic respiration processes by affecting soil moisture (SM). Liu et al. (2020) found that SM stress was most intense in semiarid ecosystems, and dominated the drought stress of global ecosystem productivity. Other studies found divergent responses in primary productivity to increasing precipitation variability in global drylands (Haverd et al., 2017; Gherardi and Sala, 2019; Hou et al., 2021). The reported responses of dryland C fluxes to warming in experiments have produced inconsistent results, including positive responses, negative responses, and no effects (Niu et al., 2008; Peng et al., 2014; Wang et al., 2019). Therefore, no consensus has been reached on the mechanisms by which climate factors affect C fluxes in drylands.

Differences in vegetation characteristics such as the leaf area index (LAI) can also affect the spatial variation in C fluxes (Pastor and Post, 1993). A study showed that with increased maximum LAI, GPP and ER increased linearly, and NEP increased exponentially in Asian ecosystems (Kato and Tang, 2008). On the other hand, researchers have found that nutrients play a key role in GPP (Vicca et al., 2012) and NEP (Fernández-Martínez et al., 2014). Previous studies have shown that in nutrient-rich ecosystems, the leaf nitrogen concentration (LNC) was high (Firn et al., 2019), which may lead to enhanced photosynthesis (Wright et al., 2004) and reduced C losses (Fernández-Martínez et al., 2018). This biological activity was found to be strongly modulated by climate factors, such as water availability in drylands (Delgado-Baquerizo et al., 2013; Ukkola et al., 2021). Although many studies have been conducted on the impact of climate, or vegetation and soil factors on ecosystem C fluxes (de Vries et al., 2014; Fernández-Martínez et al., 2017), almost no evidence has been put forth to show how climate factors affect C fluxes by affecting vegetation and soil characteristics in drylands.

**Figure 1:**
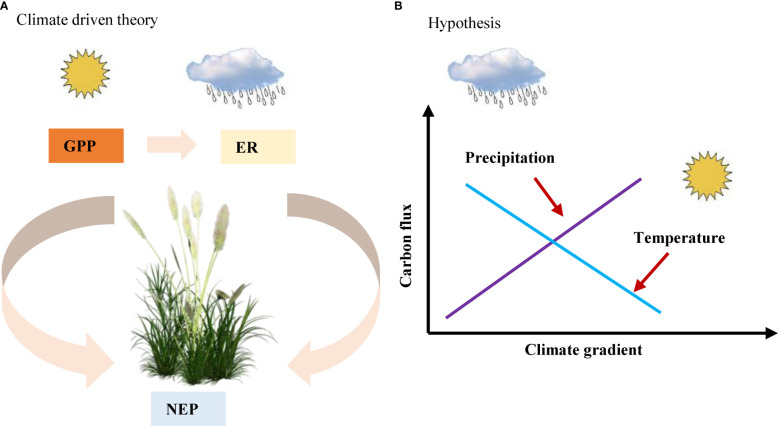
**(A)** climate driven theory and **(B)** hypothesis in our study. NEP, net ecosystem productivity; GPP, gross primary productivity; ER, ecosystem respiration.

Therefore, we aimed to assess the impact of climate (MAT and MAP), soil (SM and soil total nitrogen content (soil N)), and vegetation factors (LAI and LNC) on C fluxes and their regulatory mechanisms in Chinese drylands. Based on previous knowledge that climate factors drive spatial variation in C fluxes (**Figure 1A**), we hypothesized that climate factors, especially precipitation, may drive spatial variation in C fluxes in Chinese drylands. Moreover, with increasing precipitation, the C fluxes showed an increasing trend, while they decreased with increasing temperature (**Figure 1B**). To test our assumptions, we used the C flux data of 45 ecosystems in Chinese drylands measured using the eddy covariance method to (1) preliminarily explore how the C fluxes respond to climate, vegetation, and soil factors and (2) further analyse the regulatory mechanism of ecosystem C fluxes in Chinese drylands.

## 2 Materials and methods

### 2.1 Carbon flux data collection and screening

**Figure 2:**
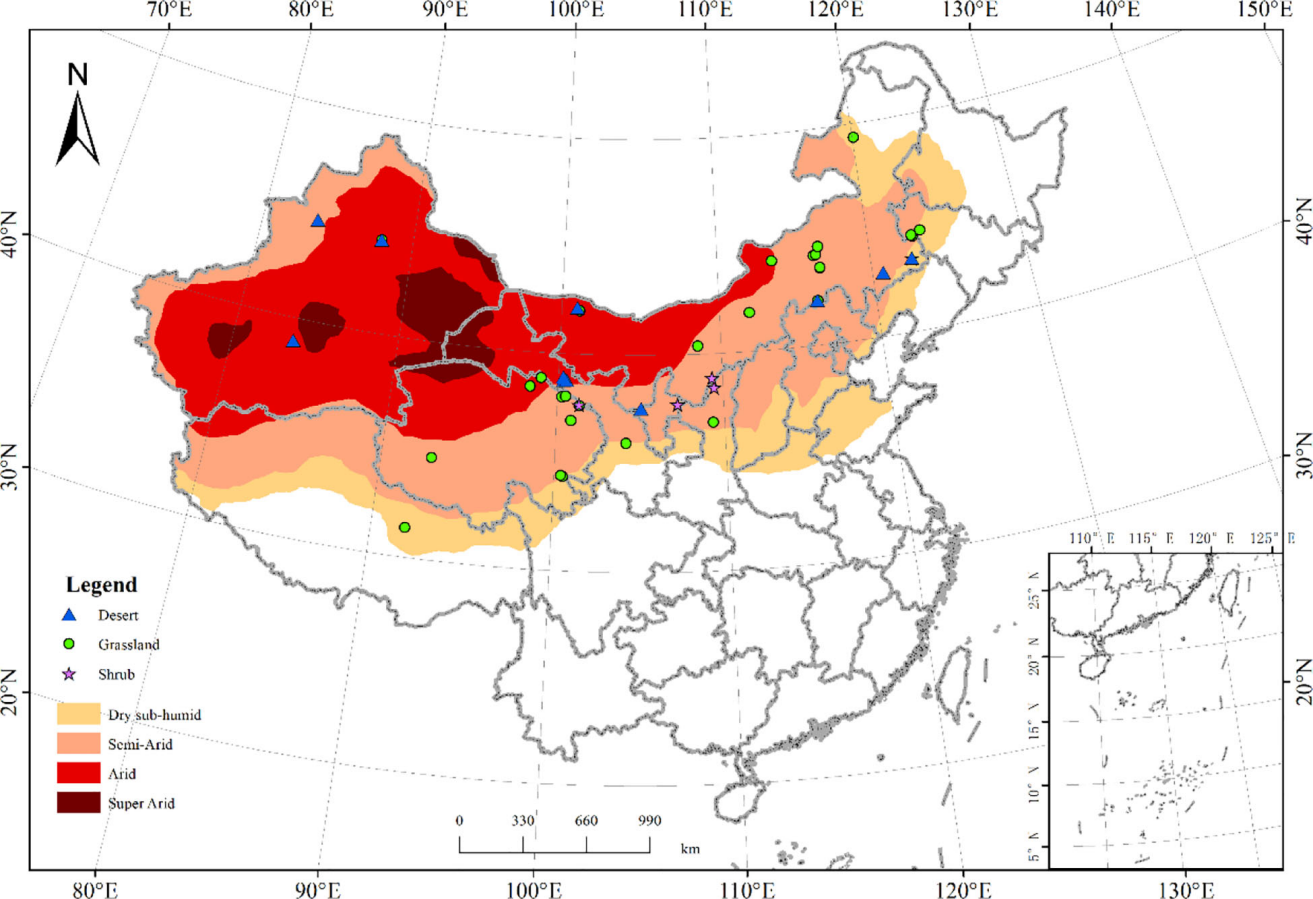
The spatial distribution of flux sites in Chinese drylands used in this study. The symbols indicate the location of the flux sites. The green circles, blue triangles, and purple pentagram symbols indicate the flux sites in the grassland (29 sites), shrub (5 sites), and desert regions (11 sites), respectively.

We collected C flux data (GPP, ER, and NEP) from the literature published on Chinese drylands over the past 18 years (2002-2020), which were measured using the eddy covariance method (LI-7200, LI-7500 et al.). All data had to be filtered and corrected at each site, using coordinate rotation, WPL correction, stored flux calculation, outlier filtering, nighttime flux correction, NEE gap filling, and partitioning. In addition, all C flux data must be measured continuously for at least one full year and located in the Chinese drylands (Chen et al., 2015). The observation years that showed abnormally high C fluxes were eliminated with three times the standard deviation, and finally, 45 C flux sites with observation durations greater than or equal to one year were selected. All C flux sites ranged from 31.37°N to 49.35°N in latitude, and 83.57°E to 123.5°E in longitude spanning the grassland (29 sites), shrub (5 sites), and desert regions (11 sites) (**Figure 2** and **Table S1**). The distribution of climate zones included a cold temperate zone, middle temperate zone, warm temperate zone, and Tibetan Plateau zone (Li et al., 2015).

### 2.2 Climate, vegetation, and soil data collection

We mainly collected climate factors including air temperature and precipitation from the same literature as the C flux data. The average air temperature and precipitation in multiple observation years were calculated as the MAT and MAP, respectively.

LAI was derived from the satellite-borne Moderate Resolution Imaging Spectroradiometer (MODIS) data product (MOD13Q1) with a spatial resolution of 1 km and a temporal resolution of 8 days from 2000 to 2018. SM was derived from Earth System Science Data (https://doi.org/10.1594/PANGAEA.912597) with a 0.1° spatial resolution from 2003 to 2018.

LNC was derived with a spatial resolution of 1 km and a temporal resolution of 8 days from 2000 to 2018 (Moreno-Martínez et al., 2018). Soil N was derived from a comprehensive and high-resolution dataset of gridded soil characteristics, with 30 arc-second spatial resolution (Shangguan et al., 2013).

### 2.3 Statistical analyses

One-way ANOVA with Duncan’s post-hoc comparisons was used to compare C fluxes across different ecosystems. The relationship between climate factors and GPP and ER was tested by linear regression. The relationship between climate factors and NEP was tested by piecewise regression, and the significance level was α= 0.05.

We detected the relationships between climate, vegetation, and soil factors, and C fluxes through correlation analysis, and the contribution of climate, vegetation, and soil factors to C fluxes was determined by using the hierarchical partitioning method (“rdacca. hp” package in R) (Lai et al., 2022).

We established a structural equation model (SEM) to assess the effects of climate, vegetation, and soil factors on C fluxes. A causal relationship was established based on previous studies on how climate, vegetation, and soil factors affect C fluxes. First, we used principal component analysis (PCA) to test for overall associations between climate, vegetation, and soil factors. Since the variables of climate, vegetation, and soil factors were closely related, PCA was conducted to create a multivariate index representing climate, vegetation, and soil groups (Wang et al., 2017). The first principal component (PC1) explained 67-92% of the total variance of each group (**Table S2**), and then the PC1 of climate, vegetation, and soil groups was used for SEM analysis. The establishment standard for the model was referenced from previous studies (Liu et al., 2023). We used Amos 21.0 (Amos Development Corporation, Chicago, IL) for SEM analysis.

All analyses were performed using R software (version 3.5.1, R Development Core Team, Vienna, Austria). ArcGis 10.1 and R were used for plotting.

## 3 Results

### 3.1 Spatial variation in carbon fluxes in Chinese drylands

**Figure 3:**
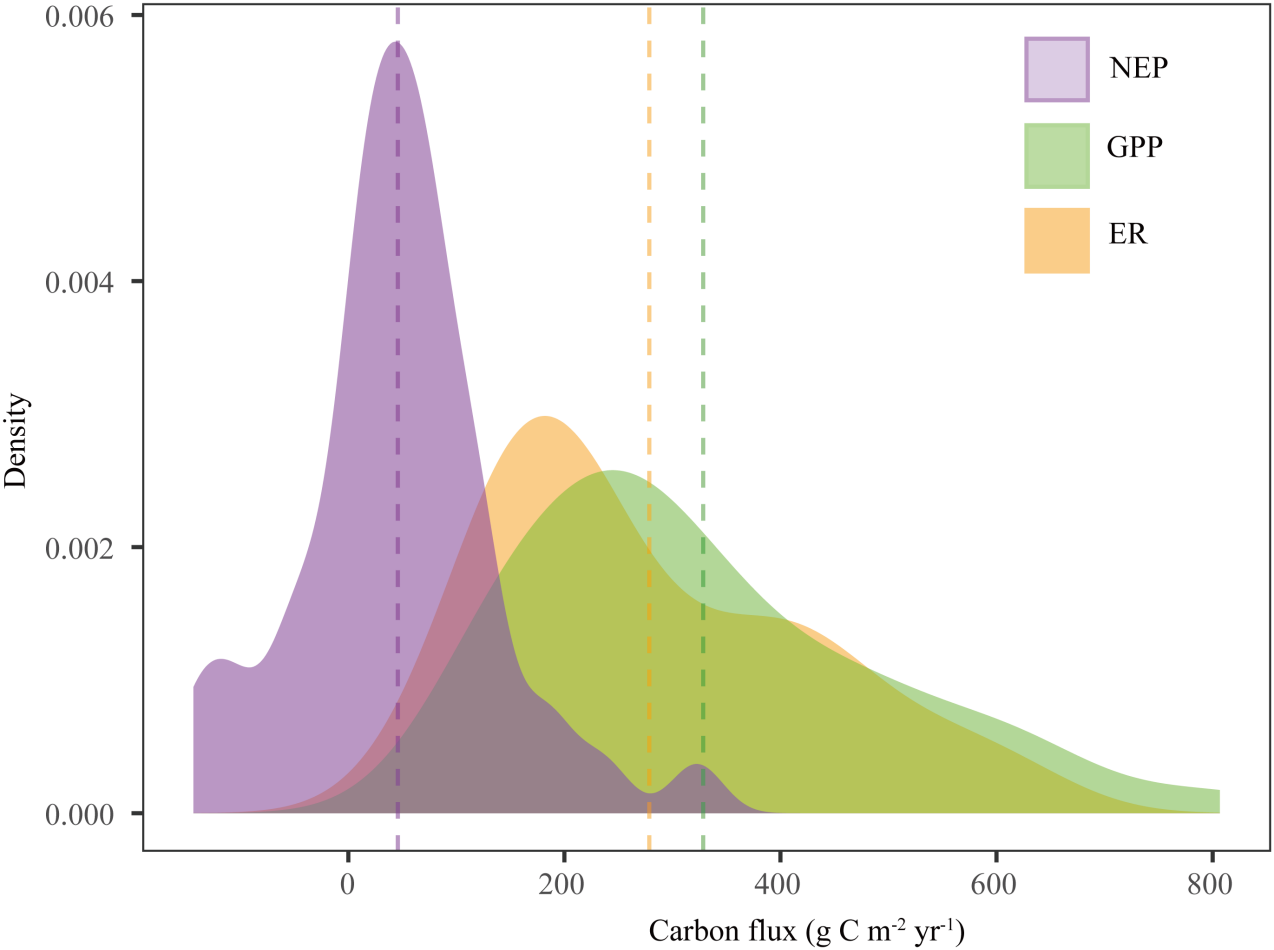
Density distributions of carbon fluxes (GPP, ER, and NEP) in Chinese drylands. The dotted lines represent the mean values of carbon fluxes. NEP, net ecosystem productivity; GPP, gross primary productivity; ER, ecosystem respiration.

The drylands in China were weak C sinks. The average GPP, ER, and NEP of the Chinese drylands were 328.54, 278.64, and 45.84 g C m^-2^ yr^-1^, respectively (**Table 1** and **Figure 3**). The ranges of NEP, GPP, and ER were -143.31-322.63 g C m^-2^ yr^-1^, 107-806.73 g C m^-2^ yr^-1^, and 62.38-630.05 g C m^-2^ yr^-1^, respectively (**Table 1** and **Figure 3**). GPP and ER showed larger variations than NEP (**Table 1**).

**Table 1:** Statistics of carbon fluxes (g C m^-2^ yr^-1^) in Chinese drylands .

Ecosystem type	Carbon flux	Mean	SE	Median	CV	Skewness	Kurtosis	Max	Min	n
Grassland	GPP^†^	342.39	35.80	278.45	1.84	0.78	-0.006	806.73	107	27
	NEP	41.76	18.65	39.60	0.42	0.62	1.44	322.63	-143.31	29
	ER	300.98	29.47	241.19	1.97	0.65	-0.8	630.05	130.63	27
Shrub	GPP	388.78	75.11	358.93	2.59	1.12	2.16	597.46	239.80	4
	NEP	60.36	13.90	68.04	1.94	-1.42	2.73	91.72	8.91	5
	ER	336.03	74.21	311.34	2.26	0.82	0.16	532.55	188.90	4
Desert	GPP	260.21	33.08	229.15	2.31	1.38	2.66	480.02	136.58	9
	NEP	50.02	25.53	44.94	0.59	-0.39	0.31	190.59	-107.85	11
	ER	186.11	31.67	187.88	1.96	0.43	-0.62	352	62.38	9
All	GPP	328.54	26.65	277.89	1.95	0.94	0.43	806.73	107	40
	NEP	45.84	13.48	43.56	0.51	0.39	1.46	322.63	-143.31	45
	ER	278.64	23.37	231.1	1.89	0.74	-0.42	630.05	62.38	40

^†^NEP, net ecosystem productivity; GPP, gross primary productivity; ER, ecosystem respiration.

For different ecosystem types in the Chinese drylands (including grassland, shrub, and desert), no significant differences in C fluxes were observed (**Table 1** and **Figure S1**). However, shrubs had the largest GPP, ER, and NEP. Although GPP and ER in grasslands were larger than those of deserts, their NEP was smaller (**Table 1** and **Figure S1**). The variation in C fluxes in shrubs was much larger than that in grasslands and deserts (**Table 1**).

### 3.2 Response of carbon fluxes to climate, vegetation, and soil factors in Chinese drylands

**Figure 4:**
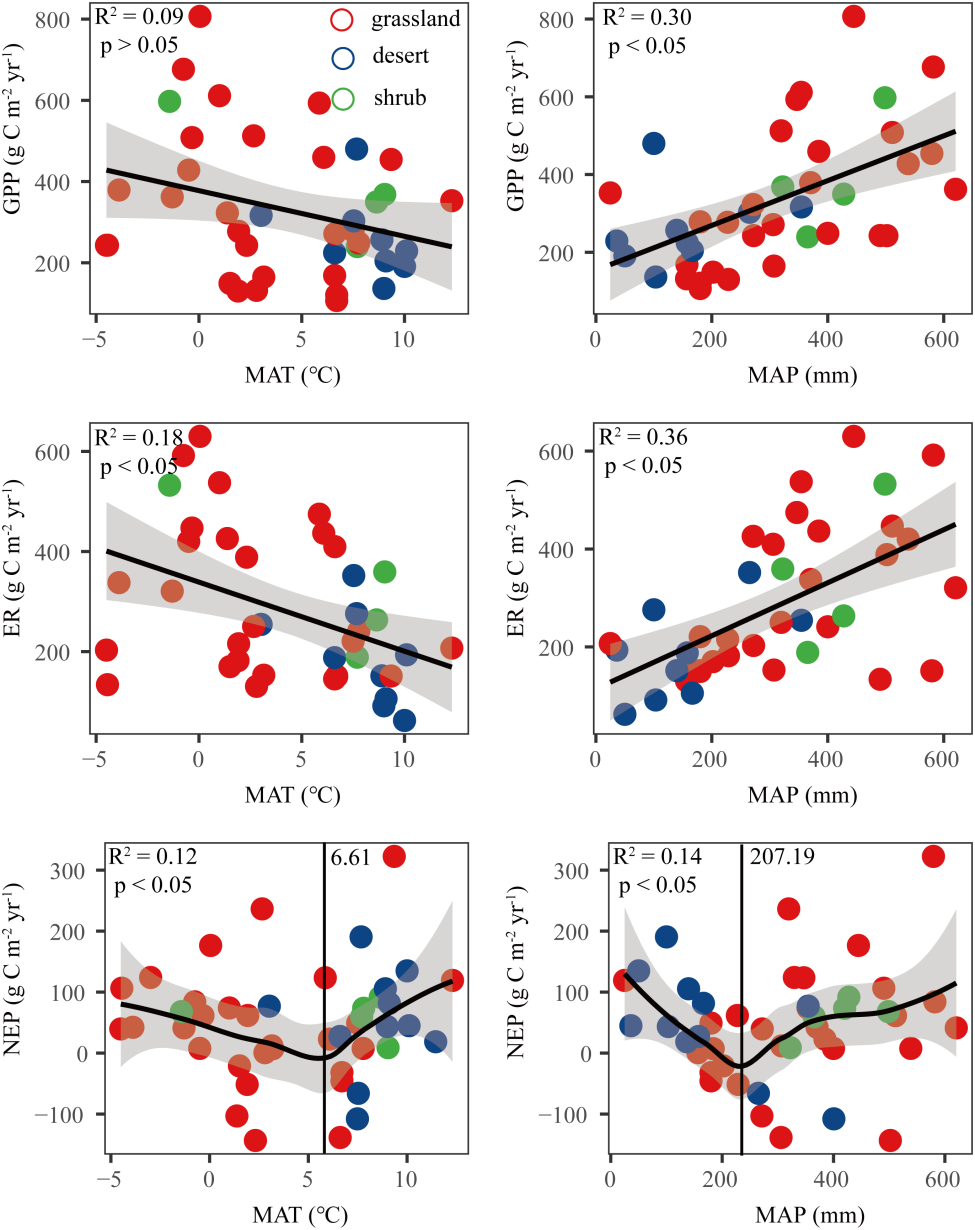
Relationships between carbon fluxes (GPP, ER, and NEP) and mean annual temperature (MAT) and mean annual precipitation (MAP). NEP, net ecosystem productivity; GPP, gross primary productivity; ER, ecosystem respiration.

The results showed that GPP and ER were positively correlated with MAP, while they were negatively correlated with MAT (**Figure 4**). The response of NEP to MAT and MAP was not linear. NEP decreased first and then increased with increasing MAT and MAP. 6.6 °C and 207 mm were the boundaries for the NEP response to MAT and MAP, respectively (**Figure 4**).

**Figure 5:**
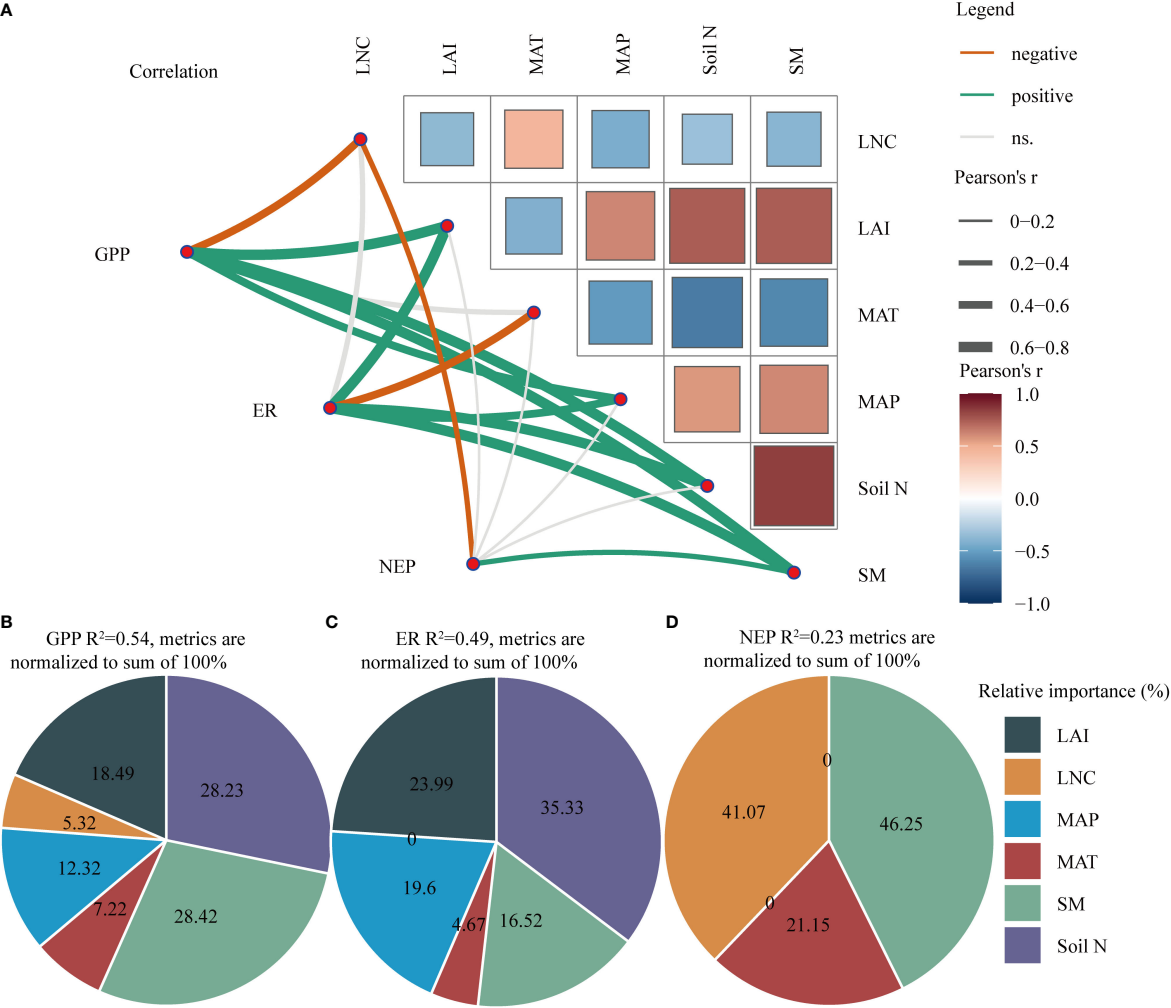
**(A)** Correlation analysis between explanatory variables and carbon fluxes (GPP, ER, and NEP). **(B–D)** Hierarchical partitioning analysis between explanatory variables and GPP, ER, and NEP. The thickness of the line indicates the strength of the correlation. The thicker the line is, the stronger the correlation. The orange line represents a negative correlation, whereas a green line represents a positive correlation. MAT, mean annual temperature; MAP, mean annual precipitation; LAI, leaf area index; Soil N, soil total nitrogen content; LNC, leaf nitrogen content; SM, soil moisture; NEP, net ecosystem productivity; GPP, gross primary productivity; ER, ecosystem respiration.

Furthermore, GPP was positively correlated with LAI, soil N, and SM and negatively correlated with LNC (**Figure 5A** and **Table S3**). ER was significantly positively correlated with LAI, soil N, and SM and was negatively correlated with MAT (**Figure 5A** and **Table S3**). However, NEP was only significantly positively correlated with SM and negatively correlated with LNC (**Figure 5A** and **Table S3**).

### 3.3 Regulatory mechanism for carbon fluxes in Chinese drylands

We found that SM and soil N were the two key factors that affected GPP, followed by LAI and MAP (**Figure 5B**). Soil N and LAI were the most important factors influencing ER, followed by MAP and SM (**Figure 5C**). However, SM and LNC had the most important effect on NEP (**Figure 5D**). Clearly, compared with climate and vegetation factors, soil factors had a greater impact on C fluxes in the drylands.

**Figure 6:**
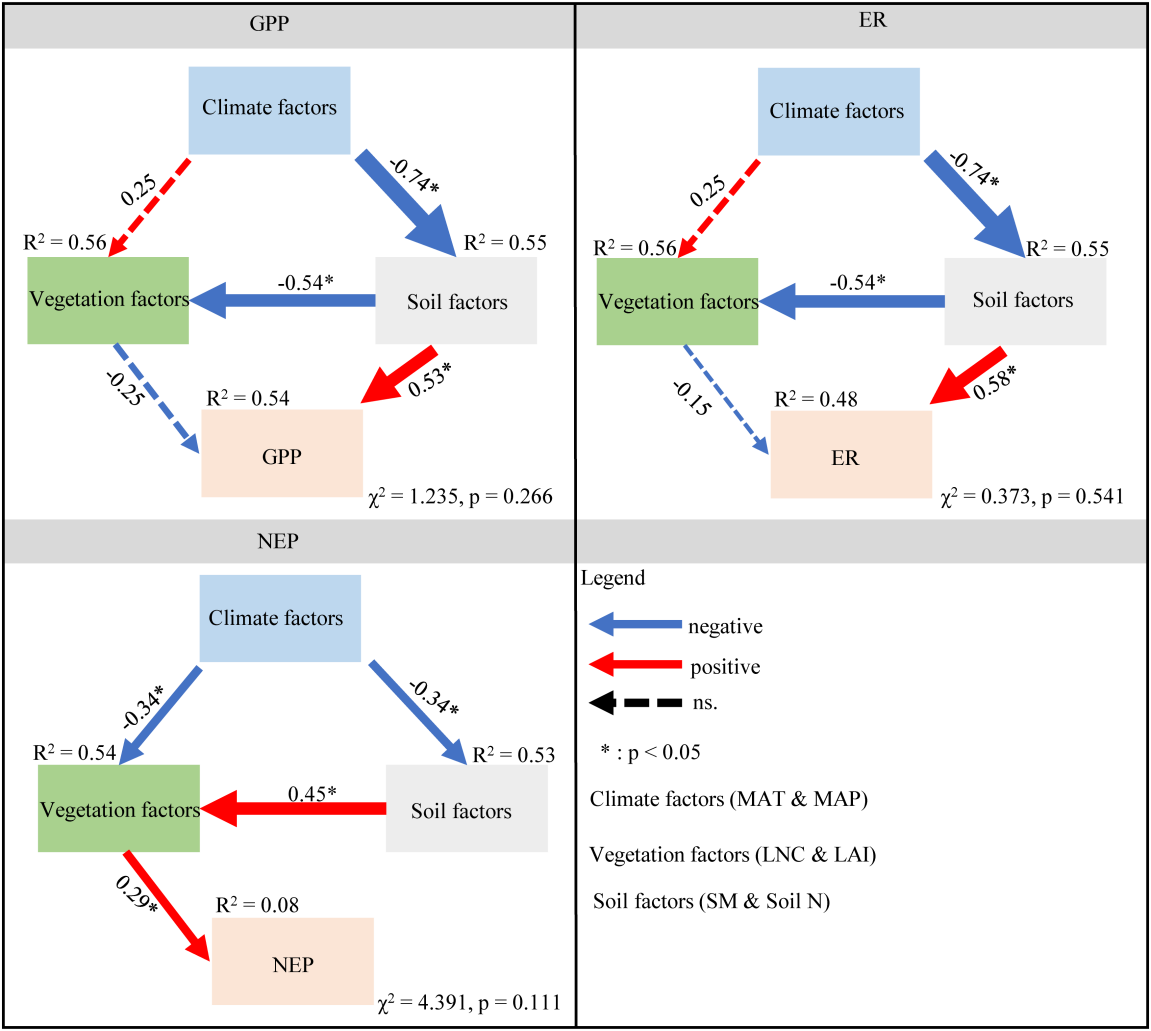
Structure equation modeling to explore the direct and indirect effects of climate, vegetation, and soil factors on GPP, ER, and NEP. The blue and red arrows indicate negative and positive relationships, respectively. The dashed line represents a nonsignificant relationship (P > 0.05). The arrow width is proportional to the strength of the relationship. The numbers adjacent to the arrows are standardized path coefficients. The proportion of variance explained (R^2^) appears alongside each response variable in the model. * indicates the significance level is less than 0.05. MAT, mean annual temperature; MAP, mean annual precipitation; LAI, leaf area index; Soil N, soil total nitrogen content; LNC, leaf nitrogen content; SM, soil moisture; NEP, net ecosystem productivity; GPP, gross primary productivity; ER, ecosystem respiration.

Soil and vegetation factors had direct effects on GPP and ER, however, climate factors had indirect effects. In summary, these environmental factors accounted for 54% and 48% of the variances in GPP and ER, respectively (**Figure 6**). Specifically, both climate and soil factors had indirect effects on NEP, whereas vegetation factors had direct positive effects on NEP. Through direct and indirect effects, soil and climate factors were the two key factors affecting C fluxes (**Figure 6**).

## 4 Discussion

### 4.1 The drylands in China are weak carbon sinks

The drylands in China were weak C sinks. The range of NEP in Asian ecosystems was wide, ranging from -150 to 1000 g C m^-2^ yr^-1^ (Chen et al., 2013), and our findings fall within this range. The range of NEP we studied was significantly larger than that of other drylands in the world (Chen et al., 2015), which may be due to the large uncertainty of C sink strength in Chinese drylands and the differences in the location and geographical range of this study. Compared with previous studies, our study sites included more extensive changes in climatic conditions and soil types (Liu et al., 2021).

Due water supply limitations, drylands are usually weak C sinks or even C sources. Large differences exist in dryland C sources and sinks among different studies (Janssens et al., 2003; Kato and Tang, 2008; Chen et al., 2013). Drylands are widely distributed, and their ecological environment is usually fragile and limited by temperature or water or both. Furthermore, the drylands were often affected by various management practices (such as grazing, harvesting, irrigation, and drainage) (Cao et al., 2004; Fu et al., 2009), which lead to large differences in the C absorption or release from drylands.

### 4.2 Response of carbon fluxes to climate, vegetation, and soil factors in drylands

Most studies have shown that GPP and ER are mainly affected by MAT and MAP, and they increase with increasing MAT and MAP (Law et al., 2002; Hirata et al., 2008; Kato and Tang, 2008; Yi et al., 2010; Chen et al., 2013; Yu et al., 2013). However, our results reveal that GPP and ER were negatively correlated with MAT in the drylands. Drought caused by warming is expected to reduce GPP and ER (Maestre et al., 2015), so with an increase in temperature, its GPP and ER showed a downwards trend. In grasslands limited by water, precipitation affects the relationship between soil temperature and respiration. Precipitation reduces the sensitivity of soil respiration to temperature, leading to a reduction in soil respiration, thereby reducing ER (Wang et al., 2014). When soil water is insufficient, the soil mineralization rate is low (Liu et al., 2021). Furthermore, rising temperatures have led to a substantial increase in the occurrence of compound warm-season droughts in drylands (Gampe et al., 2021).

**Figure 7:**
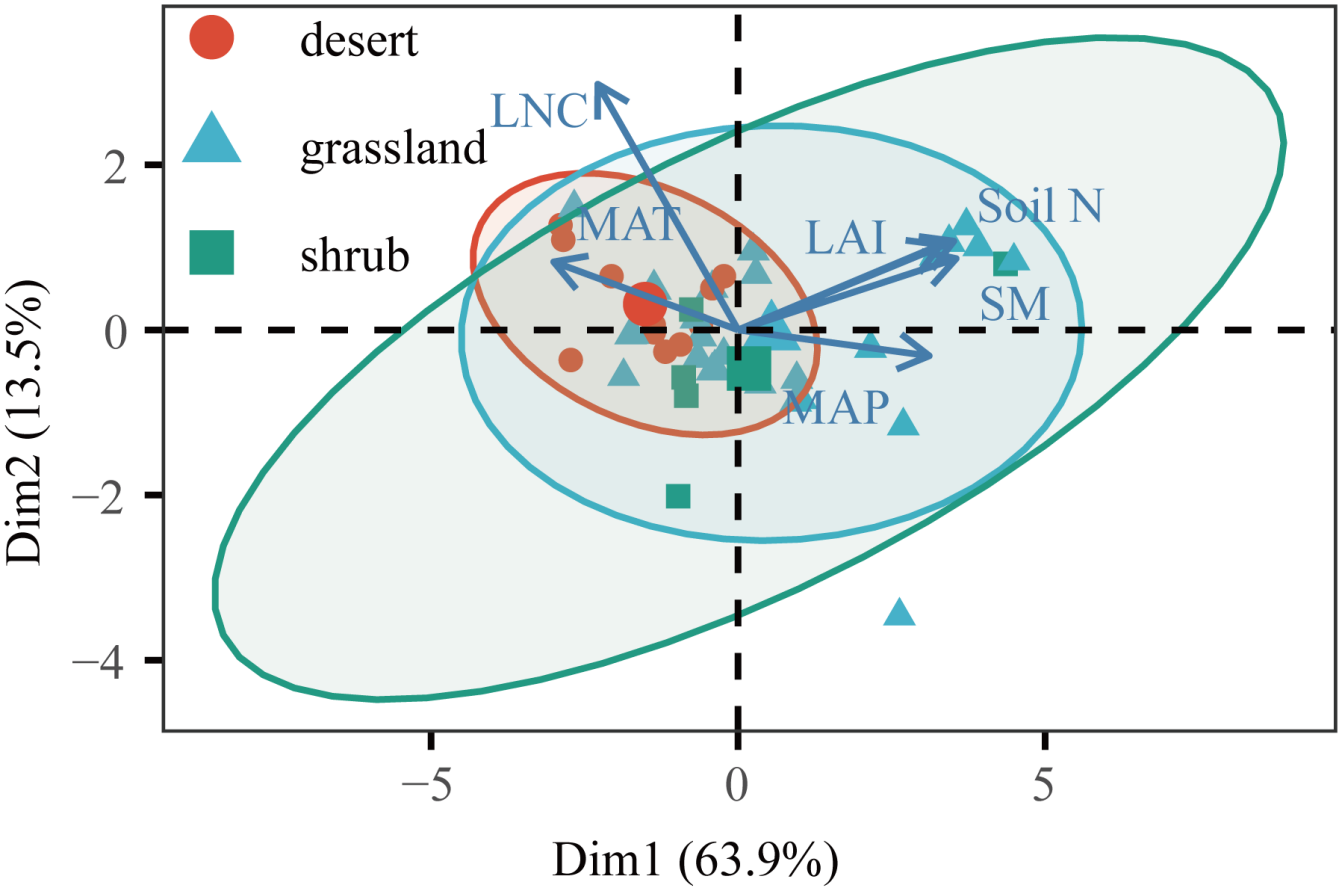
Environmental dimensions from principal component analysis (PCA) for Chinese drylands. Variables used for the PCA are displayed with their vector. The environmental factors were normalized and standardized by transforming the observations into z-scores. MAT, mean annual temperature; MAP, mean annual precipitation; LAI, leaf area index; Soil N, soil total nitrogen content; LNC, leaf nitrogen content; SM, soil moisture.

Climate determines the geographical distribution of vegetation types, and these different vegetation types can directly affect ecosystem C fluxes (Prentice, 1990; Prentice et al., 1992). Consistent with previous results (Kato and Tang, 2008), GPP and ER showed an increasing trend with increasing LAI. Surprisingly, our results showed that high LNC was associated with fewer C fluxes. Moreover, LNC had a greater influence on GPP than ER, so NEP showed the same trend (Fernández-Martínez et al., 2014). Our findings implied the colimitation of C fluxes by multiple environmental variables in drylands, and these trends may partially be explained by the interaction of MAT and LNC (**Figure 7**). We found that LNC was positively correlated with MAT (**Figures 5A**, **7**), so the C fluxes responded to LNC in the same manner as MAT. The optimal LNC increased with decreasing temperature because the photosynthetic capacity per Rubisco decreased (Muller et al., 2011), and the desert had a higher vegetation LNC (**Figure 7**). Other findings suggest that C fluxes are jointly controlled by a suite of environmental variables that covary.

Researchers have reported that permafrost melting may promote plant growth by increasing the availability of soil N (Yang et al., 2021). We detected a significant relationship between GPP and ER and soil N, which supports the claim that climate can promote plant growth by improving soil N and by enhancing the absorption of C by ecosystems. As previously reported, SM conditions also affect C fluxes (Yi et al., 2010). Our findings showed that GPP, ER, and NEP were positively correlated with MAP, but the correlation coefficient was much lower than that of SM. This is because SM dominates ecosystem productivity in drylands globally, because it is the direct water pool for plants and determines the amount of water that can be extracted by plant roots (Liu et al., 2020).

### 4.3 Regulatory mechanism of ecosystem carbon fluxes in drylands

In our study, we found that NEP first decreased and then increased with increasing MAT and MAP, and 6.61 °C and 207.19 mm were the boundaries for the NEP response to MAT and MAP, respectively. This threshold value of the NEP response to precipitation was almost the same as the boundaries between arid and semiarid areas. The threshold value of the NEP response to temperature was almost the same as the boundaries between the cold zone and the temperate zone. The mechanisms by which climate factors regulate C fluxes are very complex because of the diverse relationships between MAT and MAP patterns. In general, drought has a greater impact on GPP than ER (van der Molen et al., 2011), and when water is not a limitation, GPP and ER are co-limited by temperature or radiation in drylands (Ukkola et al., 2021). Furthermore, in the Chinese drylands, we found that with the increase in temperature and rainfall, ER was more responsive than GPP. Under joint temperature and rainfall limitations, individual increase in temperature or precipitation may not be conducive to NEP.

**Figure 8:**
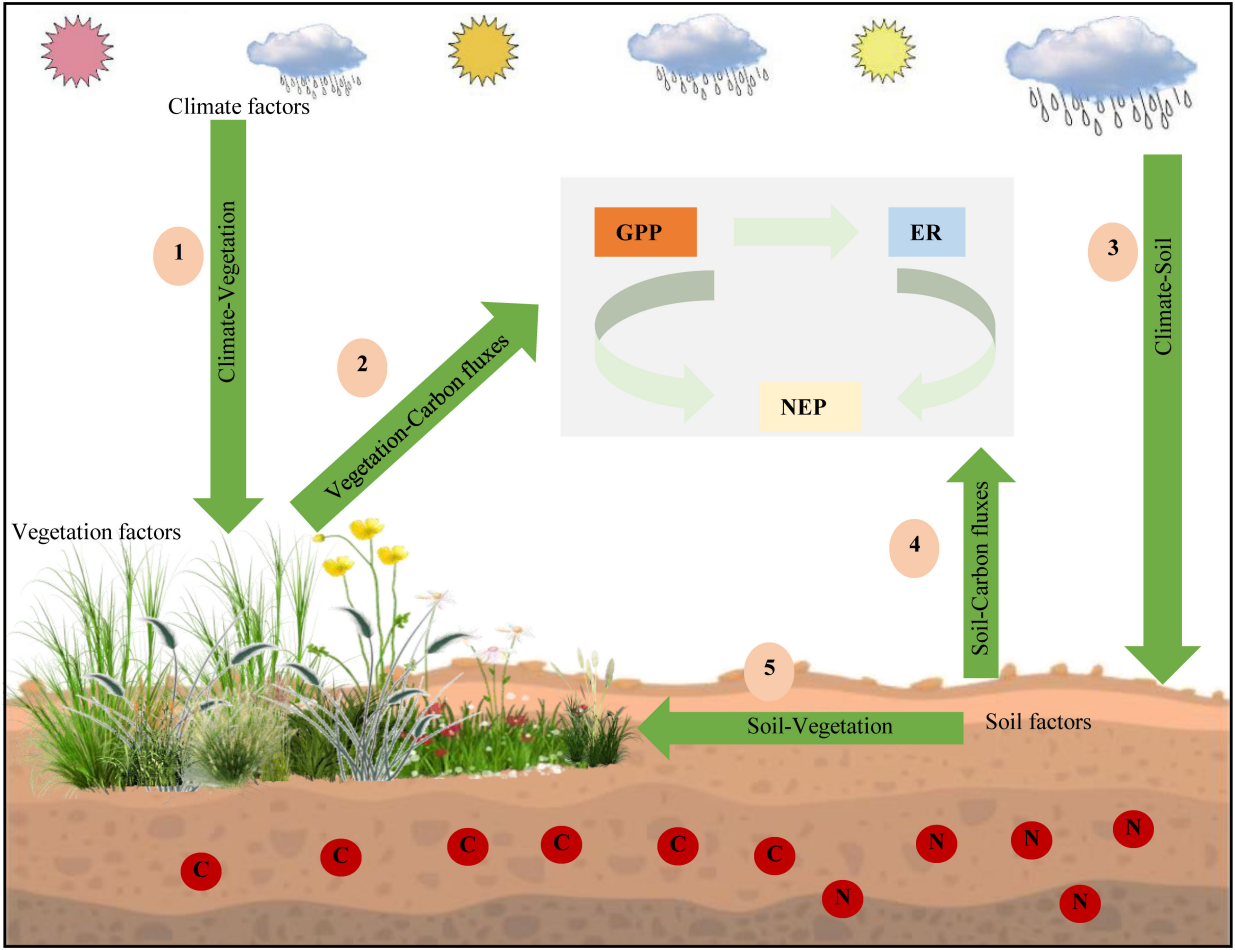
Biogeographic-ecological framework for the spatial variations in gross primary productivity (GPP), ecosystem respiration (ER), and net ecosystem productivity (NEP).

Based on the above research results, we established a simple mechanistic framework for the spatial variation in GPP, ER, and NEP in drylands (**Figure 8**). This framework mainly includes five aspects. First, we thought that changes in temperature and precipitation affected the ecosystem vegetation types. Second, these different ecosystem vegetation types had different vegetation characteristics (such as LAI and LNC), which directly affected ecosystem C fluxes. Then, we found that climate factors affected soil factors (such as SM and soil N). In addition, soil factors had the greatest direct impact on GPP, ER, and NEP. Our results showed that climate, vegetation, and soil factors had different effects on GPP, ER, and NEP. Climate factors mainly affected GPP and ER by adjusting vegetation characteristics, especially soil factors, thus affecting the variation in NEP.

## 5 Conclusion

This study examined the spatial variation in C fluxes across broad climatic and vegetation gradients in Chinese drylands. Our results provide direct evidence that Chinese drylands were weak C sinks, however, the large variation range in NEP suggests that the drylands in China have great potential to be C sinks. GPP and ER were positively correlated with MAP and negatively correlated with MAT. NEP first decreased and then increased with increasing MAT and MAP, and 6.61 °C and 207.19 mm were the boundaries for the NEP response to MAT and MAP, respectively. SM, soil N, LAI, and MAP were the main factors affecting GPP and ER. However, SM and LNC had the most important influence on NEP. Compared with climate and vegetation factors, soil factors had a greater impact on C fluxes in drylands. Our analysis showed that soil and vegetation factors had direct effects on C fluxes, while climate factors exerted indirect effects. Accounting for these different effects of climate, vegetation, and soil factors can help accurately evaluate the global C balance and predict the response of ecosystems to environmental change.

## Data availability statement

The raw data supporting the conclusions of this article will be made available by the authors, without undue reservation.

## Author contributions

ZL, GY and ZC conceived the study. ZL, WZ, TZ, LH, ZC and GY performed statistical analyses. ZL, WZ, TZ, LH, ZC and GY drafted the manuscript. All authors contributed to the article and approved the submitted version.
