# Supplementary material for: The role of climate, vegetation, and soil factors on carbon fluxes in Chinese drylands
Source: Front Plant Sci. 2023 Feb 9;14:1060066. doi: 10.3389/fpls.2023.1060066 (PMC9947249; doi:10.3389/fpls.2023.1060066)
Supplement: Supplementary file 1 [file DataSheet_1.docx]

**Table S1 Site information in this study**

| Site | Latitude  (°N) | Longitude  (°E) | MAT^†^  (℃) | MAP  (mm) | Grassland type |
| --- | --- | --- | --- | --- | --- |
| NQG | 31.37 | 91.90 | -1.30 | 620.50 | Grassland |
| SJG01 | 34.35 | 100.48 | -0.50 | 538.80 | Grassland |
| SJG02 | 34.40 | 100.40 | -0.34 | 511.32 | Grassland |
| FHG | 34.72 | 92.89 | -4.50 | 272.00 | Grassland |
| SACOLG | 35.95 | 104.13 | 7.78 | 400.04 | Grassland |
| ASG | 36.86 | 109.32 | 9.35 | 579.53 | Grassland |
| HYG | 36.95 | 100.85 | 1.00 | 354.20 | Grassland |
| SPD | 37.53 | 105.03 | 11.45 | 136.50 | Desert |
| HBG | 37.62 | 101.32 | -0.76 | 582.10 | Grassland |
| HBS | 37.67 | 101.33 | -1.43 | 498.62 | Shrub |
| YCS | 37.71 | 107.23 | 9.04 | 322.36 | Shrub |
| YKG | 38.01 | 100.24 | -4.45 | 490.35 | Grassland |
| ARG | 38.05 | 100.46 | 0.05 | 444.81 | Grassland |
| SLG | 38.42 | 98.32 | -3.89 | 370.47 | Grassland |
| YLS | 38.45 | 109.47 | 8.64 | 427.50 | Shrub |
| HZD | 38.77 | 100.32 | 9.10 | 166.40 | Desert |
| SSD | 38.79 | 100.49 | 8.90 | 139.70 | Desert |
| DSG | 38.84 | 98.94 | 2.66 | 319.60 | Grassland |
| MWS | 38.88 | 109.37 | 7.80 | 420.00 | Shrub |
| BJD | 38.92 | 100.30 | 9.00 | 103.30 | Desert |
| TZD | 38.96 | 83.65 | 10.00 | 50.00 | Desert |
| KBG | 40.38 | 108.55 | 7.50 | 180.00 | Grassland |
| SZG01 | 41.79 | 111.89 | 6.70 | 180.00 | Grassland |
| SZG02 | 41.79 | 111.90 | 6.70 | 180.00 | Grassland |
| HHD01 | 42.00 | 101.13 | 12.30 | 24.80 | Grassland |
| DLG01 | 42.05 | 116.28 | 3.02 | 354.54 | Desert |
| DLG02 | 42.06 | 116.28 | 2.32 | 502.06 | Grassland |
| HHD02 | 42.11 | 100.99 | 10.10 | 36.10 | Desert |
| NMD | 42.92 | 120.70 | 7.53 | 265.10 | Desert |
| TYD | 43.35 | 122.65 | 7.70 | 365.40 | Shrub |
| KED | 43.35 | 122.65 | 7.49 | 400.65 | Desert |
| XHG01 | 43.55 | 116.67 | 1.93 | 227.33 | Grassland |
| XHG02 | 43.55 | 116.67 | 1.51 | 202.00 | Grassland |
| XLG | 44.08 | 113.57 | 2.80 | 156.44 | Grassland |
| SNG | 44.08 | 113.57 | 3.20 | 184.00 | Grassland |
| XHG03 | 44.13 | 116.33 | 1.90 | 228.67 | Grassland |
| MDG | 44.16 | 116.49 | 3.15 | 307.16 | Grassland |
| FKG | 44.28 | 87.93 | 6.60 | 157.09 | Grassland |
| FKD | 44.28 | 87.93 | 6.60 | 157.63 | Desert |
| TYG01 | 44.34 | 122.92 | 6.07 | 384.57 | Grassland |
| TYG02 | 44.42 | 122.87 | 6.61 | 305.74 | Grassland |
| NMG | 44.53 | 116.67 | 1.38 | 271.37 | Grassland |
| CLG | 44.58 | 123.50 | 5.86 | 346.71 | Grassland |
| ABD | 44.62 | 83.57 | 7.67 | 100.00 | Desert |
| HLG | 49.35 | 120.10 | -2.99 | 329.70 | Grassland |

^†^MAT, mean annual temperature; MAP, mean annual precipitation.

**Table S2 The first principal component (PC1) explains the variance of the total variance of each group**

|  | GPP^†^ | ER | NEP |
| --- | --- | --- | --- |
| Vegetation factors | 67.15% | 67.15% | 68.59% |
| Soil factors | 92.46% | 92.46% | 92.15% |
| Climate factors | 78.37% | 78.37% | 76.33% |

^†^NEP, net ecosystem productivity; GPP, gross primary productivity; ER, ecosystem respiration.

**Table S3. Correlation analysis between explanatory variables and carbon flux (GPP, ER and NEP).**

| Carbon fluxes | Factors | r | p |
| --- | --- | --- | --- |
| GPP^†^ | LNC | -0.407 | <0.05 |
| GPP | LAI | 0.638 | <0.05 |
| GPP | MAT | -0.297 | >0.05 |
| GPP | MAP | 0.549 | <0.05 |
| GPP | Soil N | 0.682 | <0.05 |
| GPP | SM | 0.693 | <0.05 |
| ER | LNC | -0.272 | >0.05 |
| ER | LAI | 0.654 | <0.05 |
| ER | MAT | -0.417 | <0.05 |
| ER | MAP | 0.589 | <0.05 |
| ER | Soil N | 0.701 | <0.05 |
| ER | SM | 0.616 | <0.05 |
| NEP | LNC | -0.327 | <0.05 |
| NEP | LAI | 0.146 | >0.05 |
| NEP | MAT | 0.039 | >0.05 |
| NEP | MAP | 0.086 | >0.05 |
| NEP | Soil N | 0.156 | >0.05 |
| NEP | SM | 0.313 | <0.05 |

^†^MAT, mean annual temperature; MAP, mean annual precipitation; LAI, leaf area index; Soil N, soil total nitrogen content; LNC, leaf nitrogen content; SM, soil moisture; NEP, net ecosystem productivity; GPP, gross primary productivity; ER, ecosystem respiration.





**Figure S1. Variation of carbon fluxes (GPP, ER and NEP) in different ecosystems in arid and semiarid areas of China.** NEP, net ecosystem productivity; GPP, gross primary productivity; ER, ecosystem respiration.
